# Artificial Intelligence-Enabled Smartwatch Used for the Detection of Idiopathic Ventricular Tachycardia: A Case Report

**DOI:** 10.7759/cureus.42054

**Published:** 2023-07-18

**Authors:** Sumit Kumar, Arijita Banerjee

**Affiliations:** 1 Psychiatry and Behavioral Sciences, Tata Main Hospital, Jamshedpur, IND; 2 Physiology, Indian Institute of Technology (IIT) Kharagpur, Kharagpur, IND

**Keywords:** ai and robotics healthcare, smartwatches, deep learning algorithms, machine learning, idiopathic ventricular tachycardia

## Abstract

Cardiovascular disease has become a huge burden to human health. Artificial intelligence (AI)-enabled smartwatches continuously monitor the heart rate, which potentially helps to diagnose unwarranted rhythm and irregularity problems such as tachycardia, bradycardia, and fibrillation. Deep learning (DL), convolutional neural networks (CNN), and support vector machines are various modalities of AI adopted in the field of cardiology extensively, starting from a single echocardiogram to cardiac imaging. Yet, the efficacy and safety of machine learning in healthcare have always raised valid questions for the manufacturers of various healthcare devices. It is thus challenging and promising to see how AI in medicine will affect human lives in tackling various medical conditions along with global threats such as pandemics. A case report on idiopathic ventricular tachycardia (VT) detected by an AI-aided smartwatch is presented in this paper.

## Introduction

Artificial intelligence (AI) has conquered every part of our lives from food delivery to face and fingerprint recognition for personal security purposes, to the availability of smartwatches, to the rise of upgraded mobile phones, to the generation of various health-related applications. The complete diagnostic process in the healthcare industry with exponential growth in imaging technology, smart robotics, and implants thus makes it a prolific platform for AI applications. Deep learning (DL) is considered to be the next generation of machine learning. Machine learning has been adopted in the field of cardiology extensively, from a single echocardiogram to cardiac imaging.

In a noninvasive approach, algorithms are already in use with smartwatches where an electrocardiogram (ECG) sensor is readily available to carry out long-term algorithm monitoring. Nowadays, smartwatches are endowed with AI to estimate heart rate, heart rate variability, blood glucose level, blood pressure, and various other health metrics related to physical activity and fitness [1,2].

Thus, in our case report, we present a case of a 55-year-old male who had presented to the hospital clinic based on his AI-enabled smartwatch data indicating ventricular tachycardia (VT).

## Case presentation

A 55-year-old male came into the outpatient department around noon in October 2021 complaining of palpitations, sweating, anxiety, and mild dizziness for half an hour. The patient felt the need to visit a doctor immediately after his smartwatch (Fitbit Sense Health and Fitness watch, Fitbit, San Francisco, CA) detected an irregular heart rate of 164 beats per minute along with irregular rhythm and bizarre QRS wave morphology.

The patient was working in his office when he noticed the aforementioned symptoms. The patient had been diagnosed with hypertension one year previously and was on antihypertensive treatment (furosemide 40 mg once daily). The patient was a known case of asthma for 10 years and was on regular medications such as formoterol and budesonide. No shortness of breath at rest or on walking, chest pain, weakness, fever, tingling/numbness, or loss of consciousness was reported. No family history of cardiac arrhythmia, ischemic heart disease, or sudden cardiac death was noted, yet his mother had hypertension. There was no history of COVID, smoking, or alcohol intake. The patient was COVID-negative during the visit.

**Figure 1:**
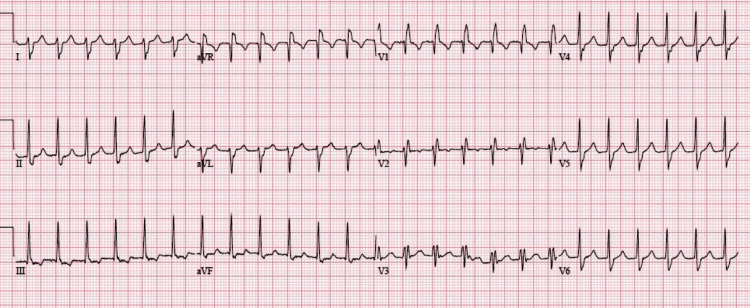
A 12-lead ECG showing features of RAD and RBBB, with ventricular rate of 166 beats per minute. ECG, electrocardiogram; RAD, right axis deviation; RBBB, right bundle branch block

Blood pressure was stable (116/80 mm Hg), and oxygen saturation (SpO_2_) was 97%. ECG was done immediately, which revealed a tachycardia of 166 beats per minute, bizarre morphology of QRS complexes with a duration of less than 130 ms, and prominent right axis deviation, indicating features of right bundle branch block as depicted in Figure 1. No signs of myocardial ischemia were noticed.

At this point, because the patient was hemodynamically stable, an adenosine intravenous drip was started for one hour, but this did not reverse the tachycardia. This finding caused the treating physicians to rule out paroxysmal supraventricular tachycardia (PSVT). The next option was electrical cardioversion with sedation. VT was reversed, and soon, the sinus rhythm was seen to be at 82 beats per minute. The initial blood investigations were done along with arterial blood gas analysis and revealed severe hypokalemia (2.3 mEq/L), whereas the rest of the parameters such as complete blood count, serum magnesium, cardiac troponin, D-dimer, prothrombin time, lipid profile, and thyroid profile were all within normal range. Hypokalemia was simultaneously reversed with intravenous potassium.

The patient was kept for 48 hours for further observation and electrophysiological studies along with coronary angiography to rule out any organic heart disease. Based on cardiologist’s advice, 24-hour Holter monitoring, echocardiography, and coronary angiography were normal, thus ruling out any structural defects in the heart or blood vessels. A diagnosis of idiopathic fascicular VT was made. Further electrophysiological study was recommended to learn the exact cause of VT, yet due to patient anxiety, the cardiologist advised him to go with oral medications first. The patient was discharged with a six-month long-term follow-up with the cardiologist rather than relying only on smartwatch. The patient was advised to take oral verapamil along with telmisartan for the control of fascicular VT, as well as hypertension.

After four consecutive visits with the cardiologist since the last episode, the patient is doing well with no further complaints. Along with his smartwatch, he makes sure to get regular checkups with a physician, along with ECG and relevant blood investigations.

## Discussion

Research in medicine has increased due to intense collaboration between AI and healthcare in the past few years. In various fields such as oncology, radiology, and robotic surgery, cardiovascular health research is one potential area that has seen immense profit from the use of various AI-enabled clinical devices in the form of health applications, smartwatches, or fitness bands, especially during the pandemic. Some of the established areas within AI are reasoning, where the algorithm is used to make use of symbolic rules, and the optimization of a problem is done to respond to the queries from the input data and machine learning that do not adhere to symbolic rules to interpret data; rather, the raw data need to be processed and interpreted to generate necessary outputs. With the help of various modalities of AI such as DL, support vector machines, and convolutional neural networks (CNN), huge data can be processed and analyzed in decreased time, thus increasing better and prompt decision-making, as well as enabling remote access to healthcare services and improvement in patient care. AI is utilized for the analysis of heart anatomy, the study and detection of congenital heart defects, and the effective analysis of ultrasound and echocardiography. AI-aided smartwatches generally take advantage of a CNN via sensors to generate real-time measurements of heart rate, blood pressure, oxygen saturation, and blood glucose. These smartwatches aid in monitoring the heart rate and thus could potentially diagnose unwarranted heart rhythm and irregularity in conditions such as tachycardia, bradycardia, and fibrillation. Various recent studies have shown a higher than 80% prediction rate by Apple smartwatches in diagnosing atrial fibrillation in subjects who later confirmed the same from 12-lead ECG analysis [3,4].

VT occurring without any apparent structural or organic heart disease is considered idiopathic ventricular tachycardia (IVT). According to the current literature, the prevalence rate of IVT is 10%, of which idiopathic fascicular left VT is very common and prevalent among males in the 30-50 years old age group. The most common mechanism is the reentrant circuit, likely occurring in some abnormal Purkinje fibers, which are generally not sensitive to vagal massage, adenosine, or beta-blockers. Sometimes, PSVT with aberrant phenomenon might respond to verapamil, but the atrioventricular dissociation in the ECG or further electrophysiological study could help in the diagnosis. It was discovered by Belhassen et al. [4] that idiopathic fascicular VT usually responds to verapamil or calcium channel blockers rather than cyclic adenosine monophosphate (cAMP)-mediated adenosine activity, which is in accordance with our evidence. The long-term management of these cases depends on the seriousness and occurrence of the symptoms. Stable patients with nonrecurrent episodes are generally put on oral calcium channel blockers. Subjects noncompliant with medications or having recurrent episodes are checked for radiofrequency ablation. In 2022, a few of the smartwatches were tested for accuracy in detecting abnormal rhythm among approximately 50 postoperative patients. With 41% sensitivity, tachycardia was detected correctly by the smartwatches in 34 subjects [5-8].

## Conclusions

AI technologies have created an immense whirl in medical research and the healthcare sector, yet questions are being raised regarding the safety and efficacy of AI systems. Idiopathic fascicular ventricular tachycardia has an excellent prognosis, provided the diagnosis is appropriately done at the right time. With regard to this, it is advisable to do extensive research further for determining the reliability of smartwatches in diagnosing arrhythmias, and at the same time, mandatory consultation with a cardiologist is warranted for early intervention in ventricular tachycardia cases.
